# Zinc Biofortified Rice Varieties: Challenges, Possibilities, and Progress in India

**DOI:** 10.3389/fnut.2020.00026

**Published:** 2020-04-07

**Authors:** D. Sanjeeva Rao, C. N. Neeraja, P. Madhu Babu, B. Nirmala, K. Suman, L. V. Subba Rao, K. Surekha, P. Raghu, T. Longvah, P. Surendra, Rajesh Kumar, V. Ravindra Babu, S. R. Voleti

**Affiliations:** ^1^ICAR-Indian Institute of Rice Research, Hyderabad, India; ^2^ICMR-National Institute of Nutrition, Hyderabad, India; ^3^Agricultural Research Station, University of Agricultural Sciences-D, Bangalore, India; ^4^Department of Plant Breeding and Genetics, AICRIP (Rice), Rajendra Agricultural University, Samastipur, India

**Keywords:** rice, biofortification, high zinc, germplasm, varieties, AICRIP, RIL's

## Abstract

Zinc malnutrition is a major issue in developing countries where polished rice is a staple food. With the existing significant genetic variability for high zinc in polished rice, the development of biofortified rice varieties was targeted in India with support from HarvestPlus, Department of Biotechnology, and Indian Council of Agricultural Research of Government of India. Indian Institute of Rice Research (IIRR) facilitates rice varietal release through All India Coordinated Rice Improvement Project (AICRIP) and also supports rice biofortification program in India. Various germplasm sets of several national institutions were characterized at IIRR for their zinc content in brown rice using energy-dispersive X-ray fluorescence spectroscopy indicating the range of zinc to be 7.3 to 52.7 mg/kg. Evaluation of different mapping populations involving wild germplasm, landraces, and varieties for their zinc content showed the feasibility of favorable recombination of high zinc content and yield. Ninety-nine genotypes from germplasm and 344 lines from mapping populations showed zinc content of ≥28 mg/kg in polished rice meeting the target zinc content set by HarvestPlus. Through AICRIP biofortification trial constituted since 2013, 170 test entries were nominated by various national institutions until 2017, and four biofortified rice varieties were released. Only the test entry with target zinc content, yield, and quality parameters is promoted to the next year; thus, each test entry is evaluated for 3 years across 17 to 27 locations for their performance. Multilocation studies of two mapping populations and AICRIP biofortification trials indicated the zinc content to be highly influenced by environment. The bioavailability of a released biofortified rice variety, *viz*., DRR Dhan 45 was found to twice that of control IR64. The technology efficacy of the four released varieties developed through conventional breeding ranged from 48 to 75% with zinc intake of 38 to be 47% and 46 to 57% of the RDA for male and female, respectively. The observations from the characterization of germplasm and mapping populations for zinc content and development of national evaluation system for the release of biofortified rice varieties have been discussed in the context of the five criteria set by biofortification program.

## Introduction

Among various food crops, rice is the world's most important crop, and more than half of the global population is dependent on rice. Greater than 90% of rice is produced and consumed in Asia (1). India is the second largest producer of rice with a production of 112.76 million metric tons during 2017–2018 (2). Imbalanced supply of essential amino acids, micronutrients, and vitamins leads to their deficiency or accumulation affecting the human metabolism. According to World Health Organization, zinc deficiency is the fifth important factor for illness and diseases in developing countries and 11th in world (3). Worldwide, the prevalence of zinc deficiency has been estimated to be ~20% in soils (4). Zinc deficiency causes diarrhea and respiratory diseases, leading to 400,000 deaths annually across the world (5, 6). Zinc deficiency is also associated with poor growth, loss of appetite, skin lesions, impaired taste acuity, delayed wound healing, hypogonadism, delayed sexual maturation, and impaired immune response (7, 8). Every year 1.31 million disability-adjusted life-years (DALYs) are lost in India because of zinc malnutrition. An ex-ante analysis of zinc biofortification of rice in India revealed that of the 1.31 million DALYs lost, 0.142 and 0.456 million DALYs could be saved in pessimistic and optimistic assumptions, if zinc-biofortified rice is introduced (9).

As a “global public good,” international agriculture research in the form of International Rice Research Institute and Center for Improvement of Wheat and Maize merged into Consultative Group on International Agricultural Research (CGIAR) initially implemented “Green Revolution” during 1960s, successfully leading to enhanced grain production through the development of high-yielding varieties (HYVs). However, grains of HYVs contain lesser amounts of nutrients; in case of rice, polishing further reduces the nutrients content, *viz*., iron and zinc. Subsequently in 1991, considering concern raised by the international nutrition community about micronutrient deficiency as a global problem, CGIAR initiated studies on developing “micronutrient-dense” staple crops (10) in the name of biofortification. In 2003, CGIAR launched HarvestPlus Challenge Program as a global program, with the objective of developing biofortified staple crops such as wheat, rice, maize, cassava, and so on, through plant breeding (11, 12). Biofortification of rice grain with iron was initiated in 1992 and zinc in 1995 (13). Improving nutrition is also being targeted under 12 of the 17 Sustainable Development Goals set by United Nations (https://www.undp.org/content/undp/…/sustainable-development-goals.html).

The word “biofortification” refers to enhancing bioavailable micronutrient content of food crops through genetic selection via plant breeding. It is a promising strategy to address nutrition security and was initiated with five criteria, *viz*., (1) crop productivity (i.e., yield) must be maintained or increased to ensure farmer acceptance; (2) the enhanced micronutrient level must have significant impact on human health; (3) the enhanced micronutrient trait must be relatively stable across various edaphic environments and climatic zones; (4) the bioavailability of micronutrients in enriched lines must be tested in humans to ensure that they improve the micronutrient status of people preparing and eating them in traditional ways within normal household environments; and (5) consumer acceptance must be tested (taste and cooking quality must be acceptable to household members) to ensure maximum impact on nutritional health (14).

Although a few independent groups have initiated research on biofortification since 2000 in India, the programs kick-started after HarvestPlus program implementation in India during 2007. Department of Biotechnology (DBT) and Indian Council of Agricultural Research (ICAR) of Government of India have also initiated support to biofortification projects leading to the consorted national and international research efforts toward the development of biofortified rice varieties. With the negative impact of zinc deficiency on human metabolism, especially in countries with rice as a major staple food, development of zinc-biofortified varieties has become one of the important objectives of crop improvement programs. The success of biofortification relies on the existence of diversity for the target trait in the germplasm of crop, successful recombination of the target trait with yield, and identification of suitable recombinants with yield and target trait through multilocation evaluation. To phenotype zinc content of rice grains across thousands of germplasm lines or mapping populations, X-ray fluorescence spectroscopy (XRF) was found to be promising because of its high throughput, cost-effectiveness, and rapid analyses over atomic absorption spectrometry and inductively coupled plasma mass spectrometry (15). HarvestPlus has supported rice biofortification research of several laboratories in India through sponsoring XRF equipment and providing preliminary biofortified rice breeding lines.

ICAR–Indian Institute of Rice Research (IIRR) is a national institute that facilitates rice varietal release through All India Coordinated Rice Improvement Project (AICRIP) (http://www.icar-iirr.org/); IIRR is also translating the benefits of the research on zinc biofortification of rice to society through release of biofortified rice varieties through AICRIP and coordination of a Consortia Research Platform on biofortification of the cereals. In the present study, we summarize our observations from the studies on characterization of germplasm and mapping populations for zinc content and development of national evaluation system, that is, AICRIP, for the release of biofortified rice varieties and its successful implementation in India.

## Materials and Methods

Two datasets were analyzed for characterization of zinc content in rice, *viz*., (1) data of zinc content from the field experiments of genotypes and recombinant inbred lines (RILs) populations developed by our group at IIRR and (2) data from analysis of the zinc content in the germplasm and RIL populations of various national institutions including IIRR at XRF facility of IIRR.

### Conduct of Field Experiments at IIRR

The field experimental material included 170 rice genotypes comprising landraces, released varieties, and derivatives of various crosses (Table 1) and 13 RIL populations developed from the six identified donors for high zinc in polished rice (Chittimuthyalu, Jalpriya, BR2655, Type-3, Suraksha, and Ranbir Basmati) and five mega varieties (MTU1010, IR64, Swarna, RPBio226, and PR116) using a single seed descent method (**Table 3**). The germplasm experiment was laid out in randomized block design with two replications. The 13 mapping populations were grown in 13 blocks in an augmented block design with IR64 and Chittimuthyalu as replicated checks. The germplasm and mapping populations were evaluated at the research farm of IIRR, Hyderabad, during the wet season following the recommended package of rice production and protection practices. The experimental soil characteristics were as follows: pH 8.2; non-saline (EC 0.7l dS/m); calcareous (free CaCO_3_ 5.01%); CEC 44.1 C mol (p+)/kg soil and medium soil organic carbon (0.69%); low soil available nitrogen (228 kg ha^−1^); high available phosphorus (105 kg P_2_O_5_ha^−1^); high available potassium (530 kg K_2_O ha^−1^); and high available zinc (12.5 mg/kg). A RIL population, *viz*., MTU1010/Jalpriya, was also grown at Agriculture Research Station (ARS), Mugad and Rajendra Agricultural University (RAU), Pusa, and another RIL population, MTU1010/Suraksha was evaluated at ARS, Mugad. For each genotype and RIL, three representative plants from the middle row were harvested at maturity, and single plant yield (g) adjusted to 14% grain moisture content was recorded. The single plant yield from the three plants was pooled and divided into three parts to be analyzed as three replicates for zinc content.

**Table 1:** Range and classification based on zinc content in brown rice of 170 rice genotypes comprising landraces, released varieties, and derivatives from various crosses.

**Name of the genotype/cross derivative**	**Zinc range (mg/kg)**	**No. of genotypes**
Chandrahasini, Manipuri, Satabdi, Bharathy, ARB-47, Varsha, Govindbhog, Jhelum, ARB-35, NDR6279 and KalluRandaikar	12–15	12
ARB-43, Karma mahsuri, Sankar, Prafulla, NDR359, PR116/NDR359, Karna, Radhi, NLR145/Sheshi, TKM-9, PR116/Sheshi, Badshabhog, Samalei, R133-968-2-1, Pushpaka, ARB-36, Sarathi, Sarabasti, PB-1, Vandana, SKAU-338, PS-4, Chandarhasini, ARB-44, Moti, NDR2008, ARB-34, ARB-40, KHP-2, Pratikhya, K-116, K-39, Jeerigesanna, Kamod, ARB-39, ARB-30, Ketakijoha, Ranbir Basmati, NDR2026, ShushkSamrat, ARB-52, ARB-54, 28(B), ARB-51, Karidaddi, ARB-46, ARB-37, 117(B), BPT5204/Chittimuthyalu-74, Naveen, Basmati 370, NC365, CharthalaiPokkali, Jodumani, ARB-48, 142(S), ChampaKhushi, ARB-50, Shalimar Rice-1, ADT45, Heera, Makom, Khitish, Type-3,Jyothi, MattaTriveni, PR116/Ranbir Basmati-63, Seetabhog, CSAR 840, PR116/Ranbir Basmati-117, Sneha and ARB-33	15.1–20.0	72
ARB-31, NLR145/Sheshi, ARB-53, Kalanamak-Birdapur, ARB-38, ARB-45, BPT5204/Vikas, BPT5204/Ranbir Basmati-72, DL-184, VytillaAnakondan, Varalu, Mahalaxmi, Pathara, Savitri, Gouri, China 1007, Panvel-3, Nuadhusara, Pratikhya, Tara, Njavara, Kalinga-III, KadamakudyPokkali, ARB-56, Sebati, Pant Sugandhdhan-17, Ranjit, Manika, Karthika, 14(S), 14-3, Kanchana, NLR 145/Type-3−42,Swarna, BPT5204/Kalanamak, Azucena, BPT5204/Jalmagna, Ambemohr, 140(M), 196(M), ARB-41, 236(K), 176(M), MSE-9, Vasumati, Pathat-23, Pimpudipasa, Tilakkachari, Aghonibora, Vasumati, 51(B), NLR 145/Type-3-70, 166(M), Moirangphou, ARB-49, 185(M), Birupa, BPT5204/Basmati 370, ARB-32, BPT5204**/**Chittimuthyalu-MS, Swarna Sub-1, RadhuniPagal, BR2655, IET-23427, 65-(S), ARB-42, Akutphou, Seetasail, PB-164, Bindli, EdavankudiPokkali, Jalpriya and Suraksha	20.1–25.0	71
Subhadra,Chittimuthyalu,ARB-55, 182(M), Sabita, Kalanamak, Taroari Basmati, 233(K), BPT5204/ Chittimuthyalu-SB, BPT5204/Sheshi, Kasturi and 24(K)	25.1–30.0	12
High Iron Rice and Improved Chittimuthyalu	30.1–35.0	2

### Evaluation of Rice Germplasm and Mapping Populations at IIRR

Seventeen sets of rice genotypes, several mapping populations, and the selected lines from mapping populations from various national institutions including IIRR were evaluated for their zinc content at the XRF facility of IIRR.

### Constitution of Biofortification Trial Under AICRIP

To evaluate the breeding lines developed with high zinc by various institutions of India, a national evaluation study was constituted under AICRIP as Biofortification trial during 2013 with two kinds of check genotypes for zinc content and yield. Initially, during 2013, to identify the promising lines in biofortification trial, a modest threshold/target zinc value of 20 mg/kg was set, as most of the cultivated rice varieties showed grain zinc content of ~15 mg/kg. From 2015, optimum threshold/target content of zinc in polished rice was increased to 24 mg/kg to match the threshold value of international target set earlier by HarvestPlus. The landraces, Kalanamak and Chittimuthyalu, with high zinc are the two check genotypes for zinc content in the trial (Supplementary Table 1). BPT5204, also known as Samba Mahsuri (BPT), popular with farmers and consumers across India because of its high yield and preferred cooking quality was included as yield check genotype. Because of the late crop duration of BPT, a mid–early duration popular variety IR64 was also included as another yield check during 2014. A hybrid (DRRH3) check was also added as another yield check during 2015 to compare the hybrids evaluated under this trial, but discontinued from 2016 because of the lack of hybrids nominated as test entries for evaluation. Under AICRIP, seed of potential lines gets nominated by the developers from various national institutions to IIRR as test entries. IIRR assigns an initial evaluation trial number to each test entry and constitutes Initial Variety Trial (IVT) with the nominated test entries along with check genotypes for yield and zinc content and sends seed along with details of the conduct of experiment to 10 to 30 locations across the country each year. After completion of the harvest, the agro-morphological and yield data of each location were sent to IIRR for compilation and identification of the promising lines with high yield and zinc. The seed is also sent to IIRR for estimation of zinc through XRF. The quality of test entries is also studied at IIRR as 16 traits/parameters (http://www.aicrip-intranet.in/PlantBreeding.aspx). The promising test entries of IVT are promoted to next year as advanced varietal trial 1 (AVT1) and subsequently to AVT2 based on their zinc content, yield, and quality. The consistent promising test entry during the 3 years of evaluation is identified as a biofortified variety.

### Zinc Estimation

The seed samples were dehusked and polished as per the standardized protocol for analyzing samples with XRF (16). Seed was cleaned manually and dehusked using JLGJ4.5 rice husker with non-ferrous components (Jingjian Huayuan International Trade Co., Ltd., Jiangsu Sheng, China) sponsored by HarvestPlus. Dehusked brown rice was sieved to remove the broken rice grains, and the full brown rice grains were cleaned with soft tissue paper. Sample was gently rubbed for 1 min against paper in the hands of a trained person wearing gloves to ensure removal of particles other than rice. Each brown rice sample was milled in a specially designed K-710 Non-Ferrous Rice Polisher (Krishi International India Ltd., Hyderabad, India), and the percentage of polishing was calculated based on weight of the polished (white) rice to the brown rice. As polished rice is the most preferred form of consumption, brown rice samples were subjected to polishing for 90 to 120 s until the desirable whiteness was reached. Cleaning of the polished rice was repeated like that of brown rice. Time gap between polishing and cleaning was minimized to avoid sticking of the bran particles to the polished grain. Each sample of brown or polished rice (5 g) was subjected to energy-dispersive XRF (ED-XRF) (OXFORD Instruments X-Supreme 8000, Highwycombe Bucks, England) sponsored by HarvestPlus at IIRR. Samples were scanned against the prestandardized method, which converts the fluorescence intensity of each sample into zinc content (mg/kg). The percentage loss of zinc was calculated using the zinc content in brown and polished rice.

### Statistical Analysis

Statistical analysis was done using R software, R foundation for Statistical computing, Vienna, Austria and all the graphs were made using ggplot2. Correlation between single plant yield and zinc content was done by performance analytics package.

### Technology Efficacy of Zinc Biofortification in Rice

The current zinc intake from rice with the popular varieties was calculated based on the per-capita rice consumption of 220 g/day (17). Under assumption that the current rice consumption pattern is maintained in India and considering the adoption of biofortified rice varieties as technology, improved intake of zinc through biofortified rice varieties was calculated. The technology efficacy (E) of zinc biofortification in rice was derived as follows:

$$\begin{array}{l} \text{Technology efficacy }\left(\text{E}\right)=\frac{\ln\left[\frac{IZ}{CZ}\right]-\left[\frac{IZ-CZ}{RDA}\right]}{\ln\left[\frac{RDA}{CZ}\right]-\left[\frac{RDA-CZ}{RDA}\right]}{\;}^{*}100 \end{array}$$

where IZ is improved zinc intake, CZ is current zinc intake, and RDA is the Recommended Dietary Allowance of zinc.

## Results and Discussion

Country-wide food supplies including those from India were found to be zinc-deficient for ~15% to 20% of the world, based on the FAO food balance sheets (18). Biofortification strategy is suggested to be the most feasible and cost-effective means of delivering micronutrients to populations with limited access to diverse diets and other micronutrient interventions (15). Of various strategies for biofortification, development of biofortified varieties is often considered the most sustainable, especially if major staple cereal food crops are targeted. Once developed, the biofortified varieties can easily be adopted by the farmers with minimal cost of cultivation, unlike agronomic biofortification with additional expenditure on external nutrient application. Biofortification of rice with zinc in polished rice is envisaged as a promising approach to address zinc deficiency in countries where rice is the major staple food crop.

### Genetic Variability for Zinc in Rice Grains

**Figure 1:**
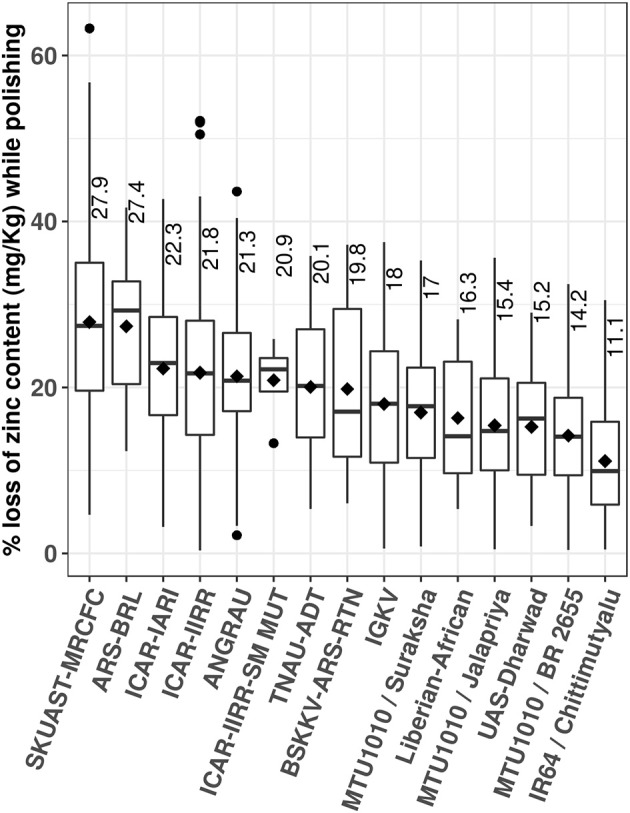
The range of loss in zinc content (%) during polishing in the germplasm sets and mapping populations. ANGRAU, Acharya N. G. Ranga Agricultural University, ARS-BRL, Agricultural Research Station, breeding lines, Shirgoan, BSKKV-ARS-RTN, Dr. BalasahebSawantKonkanKrishiVidyapeeth, Agricultural Research Station, Ratnagiri, ICAR-IARI, Indian Council of Agricultural Research–Indian Agricultural Research Institute, ICAR-IIRR, ICAR–Indian Institute of Rice Research, ICAR-IIRR-SM-MUT, Sambha Mahsuri Mutants, IGKV, Indira Gandhi Krishi Vishwavidyalaya-Raipur, SKUAST-MRCFC, Share e Kashmir University of Agricultural Science & Technology–Mountain Research Center for Field Crops, TNAU-ADT, Tamil Nadu Agricultural University, Aduthurai, UAS-Dharwad, University of Agricultural Sciences–Dharwad.

For developing varieties with high zinc in polished rice through conventional breeding, genetic variability for grain zinc content should exist in the germplasm. Since 1992, various sets of germplasm comprising varieties including *indica* and *japonica*, landraces from various countries and wild accessions, have been analyzed, and a wide variability of zinc content was reported (13, 19–21). In our study, zinc content in brown rice of the sample set of 170 rice germplasm ranged from 12 to 31.7 mg/kg (Table 1). Most of the genotypes showed zinc content in the range of 15 to 25 mg/kg, and four promising donors with >25 mg/kg zinc content were selected as donors for the development of mapping populations at IIRR. Zinc content in the germplasm comprising landraces, breeding lines, and varieties from 16 national institutions evaluated at XRF facility of IIRR ranged from 7.3 to 52.7 mg/kg (Table 2). Unlike reported mutants of IR64 with high zinc content in polished rice, higher zinc content was not observed in the mutants of BPT (ICAR-IIRR-SM-MUT) in our study (22). The mean values of zinc content in brown rice of germplasm sets ranged from 15.9 to 27.3 mg/kg. Of 3,177 germplasm evaluated, 3.1% showed zinc content of ≥35 mg/kg, and only 0.9% had zinc content of ≥40 mg/kg. Some of the reported ranges of grain zinc content include 13.5 to 58.4 mg/kg in 1,138 germplasm (13), 24.0 to 35.0 mg/kg in aromatic genotypes (14), 25.5 to 37.0 mg/kg in eight highly cultivated varieties (23), 12 to 27.6 mg/kg in 60 Iranian rice genotypes (24), and 13.3 to 34.2 mg/kg in 18 indigenous cultivars of Tripura state of India (25). In case of polished rice, zinc content ranged from 4.8 to 40.9 mg/kg in 170 rice germplasm of the present study. The reported ranges of grain zinc content in polished rice were 16.0 to 26.5 mg/kg in eight highly cultivated varieties (23), and up to 33 mg/kg zinc content in polished rice of 246 germplasm (26). Across the analyzed samples, a mean percentage loss of zinc content was found to be 19.0%, that is, 1.9 mg/kg loss in every 10 mg/kg of brown rice during polishing. The mean percentage loss of zinc varied from 11.1 to 27.9% in mapping populations and various germplasm sets (Figure 1). The reported losses of zinc content due to polished ranged from 5 to 30% (26, 27). The variation in the polishing could be due to the difference in the thickness of aleurone layer across rice genotypes as reported earlier (23, 28).

**Table 2:** Zinc content in brown rice of germplasm sets analyzed at the XRF facility of IIRR and the number of promising lines with zinc ≥35 mg/kg.

**Institution**	**No. of genotypes**	**Zinc content (mg/kg)**		**No. of genotypes with zinc content ≥35 mg/kg**
		**Range**	**Mean**	
ANGRAU	27	11.2–23.7	18.7	Nil
ARS-BRL	20	19.2–34.0	24	Nil
BSKKV-ARS-RTN	25	18.6–34.5	26.7	Nil
ICAR-IARI	221	17.0–50.2	26.9	20
ICAR-IIRR	1,317	9.6–52.7	22.7	33
ICAR-IIRR-SM-MUT	64	11.2–31.1	18.8	Nil
ICAR-NRRI	66	13.6–42.0	27.3	8
ICAR-RCNEH	51	17.4–34.2	24.8	Nil
IGKV-Raipur	840	13.1–48.3	25.1	20
Liberian-African	89	15.1–39.0	23.9	3
PJTSAU-ARS-JGL	52	7.3–28.0	15.9	Nil
RARS-AAU	73	17.0–34.8	23.1	Nil
RRS-Chinsurah	2	16.0–16.5	16.3	Nil
SKUAST-MRCFC	130	13.9–35.5	23.3	2
TNAU-ADT	47	13.9–44.3	26.8	9
UAS-Bangalore	134	9.7–36.1	22.8	2
UAS-Dharwad	19	17.4–38.7	25.7	2
Total	3,177	NA	NA	99

The zinc content in brown rice of varieties widely grown by the farmers ranged from <5 to 25 mg/kg (16, 20). The zinc content in polished rice of the popular rice varieties widely grown by the farmers ranges from (<12–14 mg/kg). Considering the threshold value of 28 mg/kg by HarvestPlus and 19.0% overall mean loss of zinc during polishing, germplasm with zinc content ≥35 mg/kg in brown rice can be considered as promising as donors to meet the target zinc.

**Figure 2:**
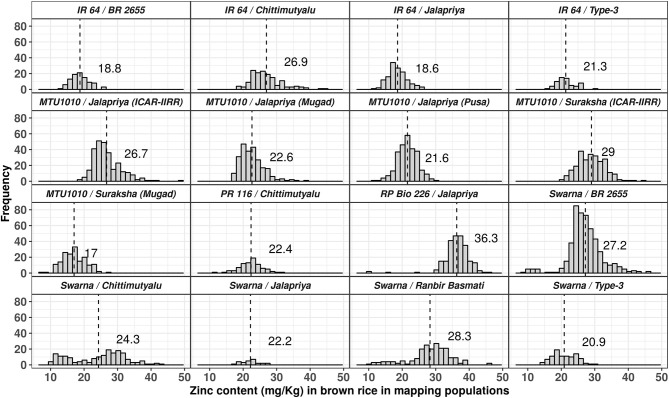
Distribution of zinc content in brown rice of 13 mapping populations evaluated at IIRR. MTU1010/Jalpriya also evaluated at ARS, Mugad, and RAU, Pusa. MTU1010/Suraksha also evaluated at ARS, Mugad.

Among 16 mapping populations grown at IIRR, the mean zinc content was least (18.6 mg/kg) in IR64/Jalpriya and highest (36.3 mg/kg) in RP Bio 226/Jalpriya (Figure 2). Of four mapping populations with IR64 as one of the parents, the mean zinc content was least (18.6 mg/kg) in cross with Jalpriya and highest (26.9 mg/kg) in cross with Chittimuthyalu. In the five mapping populations derived from MTU1010 as one of the parents, the mean zinc content was least (20.6 mg/kg) in cross with Chittimuthyalu and highest (29.0 mg/kg) in cross with Suraksha. Of the five mapping populations with Swarna as one of the parents, mean zinc content was least (19.2 mg/kg) in cross with Type-3 and highest (28.3 mg/kg) in cross with Ranbir Basmati followed closely by BR 2655. The mean zinc content of mapping populations and number of promising lines per mapping population varied despite common donors and recipients, suggesting the complexity of the trait and effect of interactions of parents for zinc content in progeny as per the cross combinations. Hence, screening of rice germplasm for higher zinc content should be a continuous process to identify compatible donors. Approximately 389 lines with zinc content ≥35 mg/kg were observed in 16 mapping populations studied (Table 3). Among 2,352 lines of mapping populations from other research groups of various national institutions analyzed using XRF facility at IIRR, 24 lines (1%) showed ≥35 mg/kg zinc content (Table 4). Thus, the development of rice breeding lines with target zinc content appears to be feasible from the recombination and selection, although the frequency of RILs with high zinc content appears to be less.

**Table 3:** Zinc content in brown rice of recombinant inbred lines (RILs) and their parents developed and evaluated at IIRR and the number of promising lines with zinc ≥35 mg/kg.

**Cross**	**No. of RILs**	**Zinc content (mg/kg)**				**No. of lines with zinc content ≥35 mg/kg**
		**P1**	**P2**	**Range**	**Mean**	
IR64/BR2655	95	9.1	24	12.4–25.3	18.8	Nil
IR64/Chittimuthyalu	151		25	18.5–44.9	26.9	11
IR64/Jalpriya	141		25	11.8–26.6	18.6	Nil
IR64/Type-3	62		19	16.3–29.9	21.3	Nil
MTU1010/Jalpriya (IIRR)	277	12	25	18.6–48.5	26.7	10
MTU1010/Jalpriya (Mugad)*	245		25	16.7–39.2	22.6	2
MTU1010/Jalpriya (Pusa)*	273		25	12.1–30.0	21.6	Nil
MTU1010/Suraksha (IIRR)	252		22	20.7–44.0	29	18
MTU1010/Suraksha (Mugad)*	169		22	6.8–27.8	17	Nil
PR116/Chittimuthyalu	85	12	25	11.5–31.8	22.4	Nil
RP Bio 226/Jalpriya	241	14	25	9.9–46.5	36.3	174
Swarna/BR 2655	495	21	24	8.2–46.2	27.2	38
Swarna/Chittimuthyalu	185		25	10.1–43.1	24.3	13
Swarna/Jalpriya	26		25	17.7–27.4	22.2	Nil
Swarna/Ranbir Basmati	202		18	9.6–45.9	28.3	20
Swarna/Type-3	98		19	14.4–29.5	20.9	Nil
Total	3,054					325

P1, Maternal parent; P2, Pollen parent. ^*^Evaluated at the Agriculture Research Station (ARS), Mugad and Rajendra Agricultural University (RAU), Pusa.

**Table 4:** Zinc content in brown rice of mapping populations and selected lines of mapping populations of various national institutions analyzed at the XRF facility of IIRR and the number of promising lines with zinc ≥35 mg/kg.

**Mapping populations (MP)/Selected lines (SL)**	**No. of lines**	**Zinc content (mg/kg)**		**No. of lines with zinc content ≥35 mg/kg**
		**Range**	**Mean**	
NS-MP	250	10.7–37.0	20.0	1
NSA-SL	30	21.3–46.5	36.3	18
NSB-MP	178	13.8–33.6	21.5	Nil
NSZ-MP	376	11.6–30.1	19.4	Nil
AMB-SL	55	12.9–31.7	19.4	Nil
IR681444/BAS-1-SL	5	13.7–31.7	19.8	Nil
SS-SL	37	11.3–31.6	18.7	Nil
NSJ, NSK, NSM, NSS, NST-MP	857	8.1–27.3	11.5	Nil
Abhaya/Moro, Abhaya/Swarna, Abhaya/TN1-SL	36	11.9–21.4	15.8	Nil
Cure-1/IR681444, Cure-10/MTU1010, Cure-2/IR681444, Cure-3/IR681444, Cure-4/IR681444, Cure-7/IR681444-SL	62	10.8–27.1	18.4	Nil
CHIR, GC, HP, IR68144/Moro-MP	317	8.3–28.8	15.7	Nil
IR681444/Kranti, IR 681444/Moro, IR681444/Abhaya, IR681444/BAS-8, IR681444/HMT, IR681444/IR64, IR681444/Kranti, IR681444/Mahamaya, IR681444/MTU1010, IR681444/Shyamala, IR681444/Swarna	73	13.8–24.5	17.7	Nil
BAS-1/IR681444, Kranthi/IR681444, Mahamaya/IR681444, Moro/IR681444, Safari-17/IR68144	25	13.5–26.9	18.8	Nil
Swarna/Bas-1, Swarna/Danteswari, Swarna/HMT, Swarna/IR64, Swarna/IR68144, Swarna/IR681444, Swarna/Moro, Swarna/Moro, Swarna/MTU1010	51	11.6–27.3	16.2	Nil
Total	2,352	NA	NA	19

Multilocation studies of two mapping populations, *viz*., MTU1010/Suraksha and MTU1010/Jalpriya, in two to three locations indicated that the zinc content to be highly influenced by environment. Earlier studies also reported significant environmental variance for zinc in rice germplasm, transgenics developed for high zinc content, and mapping populations (29–31).

**Figure 3:**
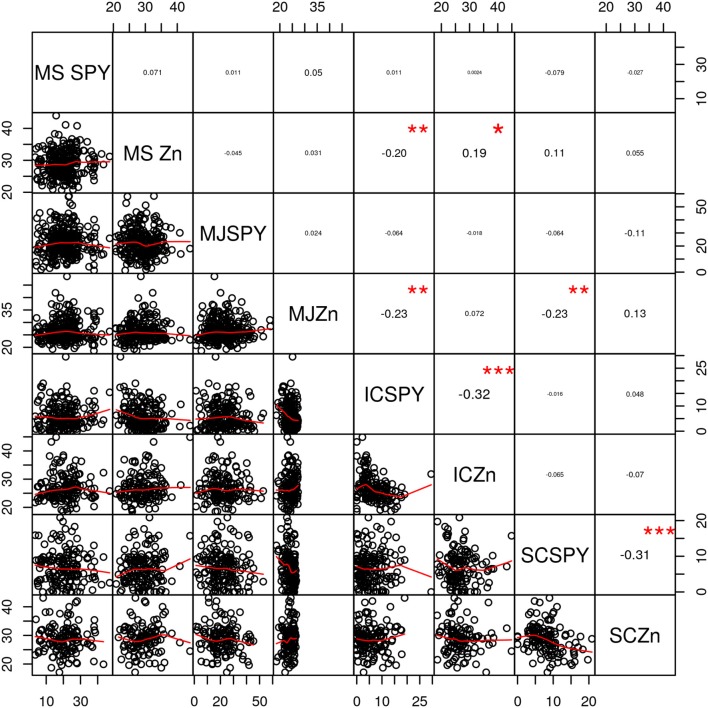
Correlation between zinc content in brown rice and single plant yield (SPY) in RILs. MS, MTU1010/Suraksha; MJ, MTU1010/Jalpriya; IC, IR64/Chittimuthyalu; SC, Swarna/Chittimuthyalu. *, **, and *** significant at the 0.05, 0.01, and 0.001 level.

Significant negative correlation (*P* < 0.001) was observed between single plant yield and zinc content in Swarna/Chittimuthyalu and IR64/Chittimuthyalu RILs, whereas no correlation was observed in MTU1010/Suraksha and MTU1010/Jalpriya (Figure 3). Overall, lines with highest zinc content showed poor yield and vice versa. While most studies report negative associations between grain zinc content and yield, a few contradicting results of positive associations were also reported (32). The dilution effect of decrease of nutrient concentrations in plant tissues with the dry matter increase is generally observed in cereals, thus explaining the inverse relationship of zinc content and yield (33). Although negative correlations are observed when the associations were studied for complete set of mapping populations, individual recombinants of high yield and zinc were also obtained. In addition to higher zinc content, the biofortified rice varieties should have yield on a par with the cultivated varieties. Until now, there is no special price for biofortified rice grains in India; thus, there is no incentive for the farmer for growing the biofortified varieties. Thus, the adoption of the biofortified varieties by the farmers is possible only when the yield is on a par with the existing popular cultivated rice varieties. Cooking quality is also important for the rice varieties for their release and adoption. Thus, for biofortified rice varieties to be released and adopted, they should possess zinc content ≥35 mg/kg without yield penalty and desired cooking quality. Nevertheless, this combination happens rarely in germplasm, and thus, conscious breeding efforts are needed to develop biofortified rice varieties with desirable attributes.

### Evaluation of Zinc Biofortified Rice Under AICRIP

With the breeding lines developed for high zinc in polished rice across the country from various projects sponsored by HarvestPlus, DBT, and ICAR, a biofortification breeding trial was initiated by AICRIP during 2013 to evaluate high zinc breeding lines without compromising yield, that is, yield must be on par the respective yield check or greater (IR64 or BPT5204). Up to 2013, the promising lines in the biofortification trial were identified with a modest threshold/target zinc value of 20 mg/kg. The optimum threshold content of zinc in polished rice since 2015 was increased to 24 mg/kg to match the international target set earlier by HarvestPlus. Considering the baseline zinc content as 16.25 mg/kg, target levels were recently revised to 28 mg/kg by HarvestPlus based on the daily requirement of the women, the consumption of rice along with absorption and retention of zinc in the body (15). However, the threshold level of 28 mg/kg still needs to be adopted by AICRIP. Since 2013, the biofortification trial has completed 5 years by April 2018 with five IVTs, four AVT 1s, and three AVT 2s (Table 5). A total of 170 entries were nominated at IVT level. Among these, 83 entries contain threshold zinc content ≥24 mg/kg, and 40 entries were found to contain desirable zinc and yield and exhibited desirable cooking quality. The 170 nominated lines represented several grain types, viz., short bold, short slender, long slender, long bold, medium slender, and medium bold and scented. From IVT, 32.3% of the test entries were promoted to AVT 1 and from AVT 1, 50.9% of the entries were promoted to AVT 2. As the AICRIP locations were categorized into seven zones, performance of each test entry is checked state-wise, zone-wise, and at country level every year. Thus, of the 170 breeding lines tested for 3 consecutive years across 5 years for their consistency in zinc content, yield, and cooking quality, only four promising test entries were recommended for their release based on their performance in one or more than one state or zones or more than one zone for 2 years to the Central Variety Release Committee (CVRC), India (Table 6, Supplementary Table 1). The promising lines in the state/s for two test entries were recommended to the State Variety Release Committee.

**Table 5:** Overview of performance of test entries in AICRIP biofortification trials of 2014–2018.

**References**	**Trial**	**No. of test entries**	**No. of test locations**	No. of entries ≥threshold zinc value^*^	**Entries with yield < yield checks^†^and poor grain quality^‡^**	**Entries with yield > yield check and acceptable grain quality**	**IET no. of released entries**
Directorate of Rice Research (DRR) (34)	IVT	26	17	12	23,814, 23,191, 23,826, 23,825, 22,624, 23,824, 23,833, and 23,834, and 23,829	23,830, 23,831, and 23,832	Nil
Indian Institute of Rice Research (IIRR) (35)	AVT 1	6	26	6	23,830, 23,831, and 23,834	23,824, 23,832, and 23,829	Nil
	IVT	41		31	23,814 (R), 24,754, 24,788, 24,768, 24,784, 24,762, 24,786, 23,826, 24,757, 24,763, 23,191 (R), 24,776, 24,756, 24,764, 24,761, 24,769, 24,755, 24,751, 24,753, 24,773	24,774, 24,766, 24,777, 24,772, and 24,783, 24,760, 24,779, 24,787, 24,771, 24,775, and 24,765	
Indian Institute of Rice Research (IIRR) (36)	AVT 2	5	22	4	23,834	23,824, 23,829, and 23,832	23,832
	AVT 1	24	19	12	Nil	24,438, 24,440, 24,779, 24,555, and 24,557, 24,760, 24,774, 24,775, 24,783, 24,360, 24,772, and 24,336	
	IVT	40		14	25,472, 25,462, and 25,466	25,475, 25,446, 25,461, 25,477, and 25,450, 25,458, 25,467, and 25,451, 25,465, 25,443, and 25,470	
Indian Institute of Rice Research (IIRR) (37)	AVT 2	12	23	8	24,779, 24,440, 24,438, 24,760, and 24,360	24,336, 24,555, 24,774, and 24,557	24,555 24,557 24,760
	AVT 1	13	24	12	25,476, 25,450, and 25,465	25,443, 25,446, 25,451, 25,458, 25,461, 25,467, 25,470, 25,475, and 25,477	
	IVT	32	21	12	Nil	25,253, 26,398, 26,385, 26,373, 26,376, 26,386, 26,396, 26,383, 26,375, 26,378, 26,380, and 26,377	
Indian Institute of Rice Research (IIRR) (38)	AVT 2	11	31	3	25,477, 25,443, and 25,475	Nil	NIL
	AVT 1	12		4	26,373, and 26,376	26,383, and 26,375	
	IVT	31	23	14	27,157, 27,158, and 27,169, 27,159, 27,155, 27,171, 27,172, 27,176, 27,177, 27,180, and 27,183	27,170, 27,169, and 27,179	

* Threshold zinc value of 20 mg/kg was set during 2013. From 2015, the optimum threshold/target content of zinc in polished rice was increased to 24 mg/kg. The same threshold values were maintained from IVT to AVT 2.

† Yield checks: IR64/BPT5204/DRRH3.

‡ *Acceptable grain quality—HRR% ≥60 with intermediate amylose content/high amylose with soft gel consistency*.

**Table 6:** Details of high-zinc rice varieties evaluated under AICRIP released through CVRC.

**IET no. and name**	**Year of release**	**Developer Institution**	**Pedigree**	**Grain type**	Zinc content in mg/kg^*^	**States**
23832 DRR Dhan 45	2016	ICAR-IIRR	IR 7307-45-3-2-3/IR 77080-B-34-3	Long slender	22.3	Andhra Pradesh, Telangana, Karnataka and Tamil Nadu
24555 DRR Dhan 48	2017	ICAR-IIRR	RP Bio 226^*^1/CSR 27	Medium slender	20.91	Uttar Pradesh, West Bengal, Kerala and Punjab
24557 DRR Dhan 49	2017	ICAR-IIRR	RP Bio 226^*^1/CSR 27	Medium slender	26.13	Gujarat and Kerala
24760 Surabhi	2017	Nuziveedu Seeds Limited	PRN-19045/PRN-14	Medium slender	22.84	Maharashtra and Gujarat

* *Threshold zinc value of 20 mg/kg was set during 2013. From 2015, the optimum threshold/target content of zinc in polished rice was increased to 24 mg/kg. The same threshold values were maintained from IVT to AVT 2 for test entries*.

### Effect of Environment on Grain Zinc Content of Rice

**Figure 4:**
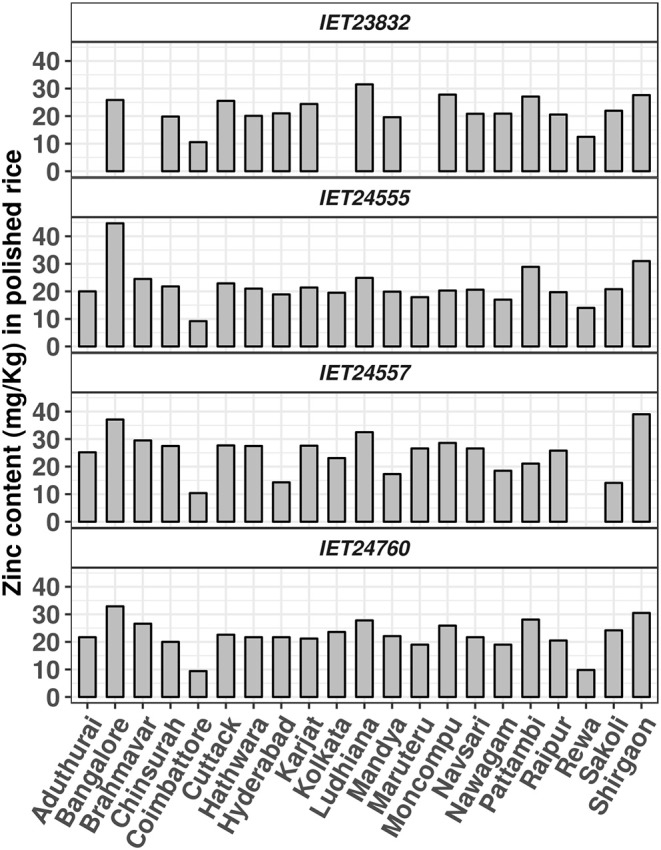
Zinc content mg/kg of zinc-biofortified varieties, released through CVRC evaluated across 21 locations under AICRIP Biofortification trial.

Although biofortified rice varieties have high grain zinc, the zinc content in the grains is highly variable, depending on the soils, seasons, agronomic practices according to AICRIP, and reported studies in India. The zinc content of two micronutrient check genotypes varied within and across locations and even within the locations across the experimental plots where IVT, AVT 1, and AVT 2 were conducted. The minimum ranges of 1.0 to 17.0 mg/kg for Kalanamak and 1.0 to 21.4 mg/kg for Chittimuthyalu were observed across 27 locations for 5 years of AICRIP studies. And maximum ranges of 9.5 to 21.4 mg/kg for Kalanamak and 12.4 to 36.3 mg/kg for Chittimuthyalu were noted (Supplementary Table 2). The grain zinc content of the four released varieties was also found to be variable with locations, as shown in Figure 4. Therefore, it is essential to identify suitable locations with favorable soils for large-scale seed production of biofortified varieties; otherwise, the potential of biofortified rice variety might not be realized because of zinc deficiency in the soil. Several countries including India, Pakistan, China, Iran, and Turkey come under zinc-deficient soils, and availability of zinc will further decrease if the soil pH is alkaline and if there is less organic matter and less moisture (3). And HYVs also take up zinc from the soil each cropping season, making the soil further deficient in zinc. Therefore, external application of zinc is essential to realize and maintain the nutrient performance potential of a biofortified rice variety.

### Biofortified Rice Varieties and Their Anticipated Impact

Considering the zinc content of the popular varieties of rice as 13 mg/kg and RDA of zinc as 12 mg/kg for male and 10 mg/kg for female (39), the zinc intake through rice consumption is 2.9 g/day per person. With the consumption of rice of existing popular varieties, the % RDA of zinc intake accounted for 24% for males and 29% for females. On the assumption that the consumption of biofortified varieties with higher zinc in polished rice will result in higher zinc intake, the effect of zinc biofortification through higher zinc intake and the extent of the improved intake to meet the RDA of zinc because of biofortified varieties in comparison to popular varieties are summarized in Table 7. With zinc-biofortified varieties, the zinc intake could account for 38 to 47% of the RDA for male and 46 to 57% of the RDA for female. The technology efficacy of zinc biofortification ranged from 48 to 67% for male and 55 to 75% for female.

**Table 7:** Technology efficacy and potential impact of biofortified zinc varieties calculated based on the zinc content of the popular varieties of rice as 13 mg/kg as baseline; RDA of zinc as 12 mg/kg for male and 10 mg/kg for female; zinc intake through rice consumption as 2.9 g/day per person and considering per-capita consumption of rice in India as 220 g/day.

**Name of the biofortified rice variety**	**Additional zinc over baseline zinc content**	**Gender**	**RDA with popular varieties (%)**	**Additional zinc with Biofortification (g/d)**	**RDA (%)**	**Efficacy (%)**
DRR Dhan 45	9.3	Male	24	4.9	41	54
		Female	29		49	61
DRR Dhan 48	7.9	Male	24	4.6	38	48
		Female	29		46	55
DRR Dhan 49	13.1	Male	24	5.7	47	67
		Female	29		57	75
Surabhi	9.8	Male	24	5.0	42	56
		Female	29		50	63

With rice as the sole diet component, in order to meet the RDA of zinc, polished grains should have 54.5 to 68.2 mg/kg zinc (without including bioavailability status) (40). Transgenic rice lines with soybean ferritin gene were developed for enhanced iron content in polished rice grains, but the same lines also accumulated higher amount of zinc ranging from 34.9 to 55.5 mg/kg (41). NASFer-274 event of IR64 transgenic rice with nicotianamine synthase (OsNAS2) and soybean ferritin (SferH-1) genes showed 45.7 mg/kg zinc content in polished rice without yield penalty or altered grain quality (31). Although zinc content of transgenic rice lines can meet the RDA of zinc, the transgenic rice lines are still to be accepted and adopted in India.

Our learning experiences since 2013 from the studies on characterization of germplasm for zinc content, development of national evaluation system for the release of biofortified rice varieties, and evaluation of biofortified rice breeding lines have been consolidated with reference to the five criteria set by the biofortification program (14).

As per criterion 1 (crop productivity, i.e., yield must be maintained or increased to ensure farmer acceptance), the recombination of zinc content and yield appears to be feasible as evidenced from the nominations made by several national research groups to biofortification trials and the release of four biofortified rice varieties through AICRIP.

Criterion 2 (impact of the enhanced micronutrient level on human health) has been indirectly confirmed through *in vitro Caco-2* cell model studies of biofortified rice varieties. Because *in vivo* human clinical tests are expensive, labor-intensive, and subject-dependent response with, results of *in vitro* bioavailability studies can be explored as a possible alternative as proof of concept for zinc-biofortified rice varieties popularization. Bioavailability of biofortified rice variety with higher zinc in polished rice showed higher absorbed zinc through *in vitro Caco-2* cell model and suckling rat pups studies (42). Preliminary studies of Indian Council of Medical Research–National Institute of Nutrition (ICMR-NIN) Hyderabad demonstrated that the zinc content and bioavailability from DRR Dhan 45 rice variety were almost twice those of the control IR64 (43). The clinical trial of one of the released biofortified rice varieties is under consideration by the Government of India.

According to criterion 3 (the enhanced micronutrient trait must be relatively stable across various edaphic environments and climatic zones), only the consistent genotypes across the states/zones and years are being released as biofortified zinc varieties under AICRIP in India. Multilocation data of AICRIP or independent research groups (31) indicated that the zinc content across the locations is highly variable and dependent on soil factors (Figure 4). Considering the wide diversity of agroclimatic regions of India, for the identification of the promising lines and their release as varieties, the data collected under AICRIP are being analyzed as performance state and zone-wise for the identification of stable varieties.

Criterion 4 addresses the bioavailability of micronutrients and is being carried out at ICMR-NIN. The coupled *in vitro* digestion/*Caco-2* cell model studies with extrinsic ^65^Zn isotopic labeling demonstrated higher absorption of zinc from DRR Dhan 45 rice in intestinal cells. The proximate composition of DRR Dhan 45 and IR64 varieties remained similar, except that DRR Dhan 45 rice had a little higher protein. Detailed studies of zinc absorption of rice genotypes with differential zinc using coupled *in vitro* digestion/*Caco-2* cell model studies are in progress. The overall food composition dictates the fate of intestinal absorption of zinc in diet. For zinc, phytate, fiber (cellulose, hemicellulose, lignin), oxalic acid, lectins, and heavy metals act as antinutrients, whereas organic acids (ascorbate, fumarate, malate, and citrate), long-chain fatty acids, β-carotene, and some of the amino acids act as pronutrients (44). As per criterion 5, consumer acceptance by taste and cooking quality is also taken care through a set of approved laboratory experiments in AICRIP coupled with panel test at IIRR.

## Conclusions

Zinc deficiency, especially in the developing countries with rice as a major staple food, is being addressed by the development of biofortified rice varieties with high zinc in polished rice with the national and international support in India. Germplasm screening for their zinc content in brown and polished rice using ED-XRF revealed wide genetic variability, and evaluation of mapping populations indicated the possibility of favorable recombination of high zinc content and yield. Although only 2.4% biofortified varieties were released out of the total test entries until 2017, the rigorous screening through AICRIP biofortification indicated the achievability of combining target zinc content, yield, and quality parameters. A preliminary study of bioavailability of a released biofortified rice variety was found to be twice that of control variety IR64. The technology efficacy and the enhancement of RDA calculated with the four released varieties were found to be promising. Thus, the benefits of biofortification research are being translated to the society as biofortified rice varieties with high zinc, hoping for their adoption and impact in achieving the nutritional security of the needy.

## Data Availability Statement

All datasets generated for this study are included in the article/Supplementary Material.

## Author Contributions

DS: analyses and writing of the manuscript. CN: planning and writing of the manuscript. PM, PS, and RK: evaluation of mapping populations. BN: economic impact of biofortification. KSum: writing of the manuscript. LR: AICRIP analyses. KSur: editing of the manuscript. PR and TL: bioavailability studies. VB and SV: Coordination of the study.

### Conflict of Interest

The authors declare that the research was conducted in the absence of any commercial or financial relationships that could be construed as a potential conflict of interest.

## Abbreviations

ED-XRF: Energy Dispersive X-ray fluorescence spectroscopy
RIL: Recombinant Inbred Line
ICAR: Indian Council of Agricultural Research
ICMR: Indian Council of Medical Research
IIRR: Indian Institute of Rice Research
AICRIP: All India Coordinated Rice Improvement Programme
NIN: National Institute of Nutrition
CRP: Consortia Research Platform
ARS: Agricultural Research Station
IVT: Initial Variety Trial
AVT: Advance Varietal Trial.
